# Hypoxia in Head and Neck Cancer in Theory and Practice: A PET-Based Imaging Approach

**DOI:** 10.1155/2014/624642

**Published:** 2014-08-21

**Authors:** Loredana G. Marcu, Wendy M. Harriss-Phillips, Sanda M. Filip

**Affiliations:** ^1^Department of Physics, Faculty of Science, University of Oradea, 410087 Oradea, Romania; ^2^School of Chemistry and Physics, University of Adelaide, Adelaide, SA 5000, Australia; ^3^Department of Medical Physics, Royal Adelaide Hospital, Adelaide, SA 5000, Australia

## Abstract

Hypoxia plays an important role in tumour recurrence among head and neck cancer patients. The identification and quantification of hypoxic regions are therefore an essential aspect of disease management. Several predictive assays for tumour oxygenation status have been developed in the past with varying degrees of success. To date, functional imaging techniques employing positron emission tomography (PET) have been shown to be an important tool for both pretreatment assessment and tumour response evaluation during therapy. Hypoxia-specific PET markers have been implemented in several clinics to quantify hypoxic tumour subvolumes for dose painting and personalized treatment planning and delivery. Several new radiotracers are under investigation. PET-derived functional parameters and tracer pharmacokinetics serve as valuable input data for computational models aiming at simulating or interpreting PET acquired data, for the purposes of input into treatment planning or radio/chemotherapy response prediction programs. The present paper aims to cover the current status of hypoxia imaging in head and neck cancer together with the justification for the need and the role of computer models based on PET parameters in understanding patient-specific tumour behaviour.

## 1. Introduction

### 1.1. The Challenge of Hypoxia in Head and Neck Cancer

Tumour hypoxia remains one of the major causes of treatment failure in solid cancers. Advanced head and neck carcinomas are often aggressive and commonly hypoxic, features that are associated with treatment resistance to both radio- and chemotherapy and also poor survival [1]. Additionally, there is evidence that hypoxia limits the effectiveness of surgery as well [2]. Studies undertaken on head and neck squamous cell carcinomas have concluded that the median partial oxygen pressure (pO_2_) is one of the strongest independent prognostic factors of both disease-free and overall survival in these patients, regardless of treatment modality [1, 3].

Hypoxia was shown to promote angiogenesis and distant metastases [4, 5] processes that add to the challenge of managing hypoxic solid tumours. Furthermore, a bell-shaped relation was found between the microvascular density of head and neck tumours and patient survival, meaning that both very low and very high concentrations of blood vessels are associated with poor prognosis [6]. Therefore, low oxygenation status and intensified angiogenesis are equally linked to treatment failure in head and neck cancer.

As a response to cell loss during treatment, head and neck tumour cells activate various mechanisms to trigger tumour repopulation. Similarly to tumour cells, endothelial cells, which form the lining of blood vessels, have also exhibited high activity during treatment, leading to regeneration of microvessel density [7]. The process of tumour repopulation and/or vascular regeneration in head and neck cancer during radiotherapy creates a vicious circle that is difficult to interrupt, which is the reason why these occurrences are commonly associated with treatment failure.

### 1.2. Hypoxia as a Promoter of Angiogenesis

Tumours need oxygen and nutrients to survive and thrive. Tumours that outgrow their own vasculature lack oxygen supplies and therefore they start creating their own vascular network to allow the oxygen transport. In fact, there is experimental evidence showing that the induction of new blood vessel formation (i.e., angiogenesis) is activated well before the tumour outgrows its vasculature [8]. Tumours exceeding the size of 1 mm^3^ are reliant on blood supply from the newly created vascular network [9].

Once the angiogenic switch is turned on, new capillaries start to sprout and generate a whole new vascular network. The newly formed vessels, however, are abnormal looking and present with leaks, shunts, and blind ends, which can often be obstructed. These obstructions cause further problems, as oxygen cannot reach the cells in the affected areas. Consequently, temporary hypoxia arises which renders the cells resistant to treatment. This type of hypoxia, also known as acute hypoxia, is one of the biggest challenges in the management of malignant neoplasms as they are both spatially and temporally unstable. In other words, it is impossible to predict where, when, and for how long a specific tumour region will be hypoxic. This fact has further repercussions on treatment planning and delivery, as the hypoxic areas based on pretreatment images might not coincide with the hypoxic areas that are present during treatment.

Chronic hypoxia, on the other hand, is more predictable, as it typically occurs in the core of the tumour and the reoxygenation process befalls during fractionated radiotherapy.

### 1.3. Hypoxia as a Promoter of Distant Metastases

Tumour hypoxia has been shown to play an essential role in the promotion of distant metastasis [4] given that the hypoxia-inducible factor-1*α* (HIF-1*α*) regulates several processes along the metastatic pathway.

Head and neck cancer metastasizes predominantly to regional lymph nodes. Both large and small tumours have the capacity to metastasize given that the primary tumour expresses the gene for metastatic spread. For instance, increased gene expression levels of the glucose transporter protein Glut-1 were found to be correlated with local recurrence, regional lymph node metastases, and poor survival in patients treated for oral squamous cell carcinomas [10]. Similar results were achieved by Zhou et al. in head and neck cancer patients, indicating a strong relationship between elevated Glut-1 levels and poor prognosis [11]. However, the risk of distant metastases was shown to be lower in head and neck tumours treated with hypoxia modifiers [12].

With today's technological advances, locoregional control of several types of cancers, including head and neck, has improved. The challenge, however, remains controlling the metastatic spread and thus in the management of the systemic disease. Despite the fact that tumour metastases are the most common cause of death among cancer patients, methods to specifically target this process are scarce. Alongside hypoxia, the metabolic information supplied by PET imaging offers valuable assessment of tumour metastases. Such information allows for further treatment planning materialized by either chemotherapy or targeted radioimmunotherapy [13, 14].

## 2. Common Assays for Tumour Oxygenation Status

Over the decades, several methods, including a variety of imaging modalities, have been trialled to detect hypoxia and differentiate between the acute and the chronic type, whenever possible. Hypoxic subvolumes within a tumour need adequate considerations during treatment planning and delivery; thus they need to be reliably identified. Due to large heterogeneities in tissue oxygenation and interpatient variations [15], hypoxia needs to be individually assessed for each patient for an accurate target definition and identification of hypoxic subvolumes for dose painting.

Despite all efforts to develop pretreatment assays to evaluate the association between the level of hypoxia and treatment outcome, several predictive assays failed the* in vivo* testing [40]. The main objective of predictive assays for tumour oxygenation status is to select the likelihood of benefit from the addition of hypoxic cell sensitizers, hypoxic cytotoxins, or other adjuvant therapies that would lead to an increased therapeutic ratio in hypoxic tumours. Nowadays, the importance of microvessel density assessment increases, together with the development of antiangiogenic agents and their inclusion in treatment protocols for patients who might benefit from these therapies.

Both direct and indirect methods have been developed in order to evaluate and quantify the level of oxygenation in tumours (Table 1). The most common methods involve (1) the use of polarographic electrodes, (2) immunohistochemical staining of hypoxic cells, and (3) a less invasive approach—PET imaging using hypoxia-specific radiotracers.

It has already been mentioned that microvessel density is a good predictive factor of treatment outcome in head and neck cancer [6]. The evaluation of vascular density represents an indirect method to determine tumour oxygenation and is undertaken using tumour biopsies. This technique originated in the 1960s, when Kolstad showed that tumours with long intercapillary distances (i.e., with poor vascular networks) had low oxygenation and higher probability to recur than tumours with short intercapillary distances [41]. Microvascular density measurements involve immunohistochemical techniques for counting of vessels that were previously labeled with endothelium-specific markers. One drawback of this method is the lack of information on acutely hypoxic cell populations. However, vascular density measurements offer indications regarding chronic hypoxia, demonstrating that chronically hypoxic cells significantly contribute to the overall hypoxic fraction and also influence treatment outcome.

One of the most widely used tools for tumour oxygenation measurements (oxygen tension or pO_2_) is the polarographic oxygen electrode. The principle of this technique is based on the polarographic reduction of molecular oxygen at a platinum electrode, which creates an electric current. The magnitude of the current depends on the oxygen quantities that reach the electrode. The oxygen electrode that is currently used in labs is manufactured by Eppendorf-Netheler-Hinz GmbH, Hamburg, Germany. Due to its good correlation with the clinical outcome, the Eppendorf electrode is still considered the gold standard in determining the oxygen tension [42].

One advantage of this technique is that allows quantitative assessment of oxygen distribution levels within the tumour, without, however, distinguishing between necrotic and viable cancerous tissues. A main drawback is linked to the invasive approach, which limits the technique to superficial tumours only.

Functional imaging techniques, besides having other advantages, are noninvasive methods for the assessment of tumour metabolism and of various tumour-related kinetic parameters. Nowadays, tumour hypoxia is successfully evaluated using such imaging techniques. Positron emission tomography (PET) and blood oxygen level-dependent magnetic resonance imaging (BOLD MRI) are two diagnostic imaging methods widely used for oncologic investigations. These two robust techniques are characterized by ease of use and reliability and are able to assess the heterogeneous distribution of oxygen with the tumour [43].

BOLD MRI uses paramagnetic deoxyhaemoglobin as contrast agent (to target red blood cells), which makes the method sensitive to pO_2_ in blood vessels and also in the neighbouring tissues. However, the technique does not allow direct measurement of tissue pO_2_. There are various studies looking into the relationship between pO_2_ and BOLD signal changes to find a reliable correlation between these parameters [44, 45]. Given that in acute hypoxia the poorly oxygenated regions extend up to the vasculature, BOLD MRI seems to be a more sensitive measure of oxygen levels for perfusion-related hypoxia than for diffusion-dependent or chronic hypoxia. Due to the large distance between chronically hypoxic areas and the red blood cells from existing tumour vessels, BOLD MRI cannot reflect the status of chronically hypoxic tumour regions [43]. While this MRI technique does not provide a quantitative measure of pO_2_, there is experimental evidence showing an association between variations in tumour oxygenation and changes in BOLD signals (spin lattice relaxation rate) [44].

Of all the currently existing techniques for* in vivo* detection of hypoxia, positron emission tomography is the best validated and the most used clinical method. PET is a noninvasive, functional imaging technique, which measures the* in vivo* distribution of radiolabelled isotopes, after the injection of a radioactive contrast material. For hypoxia imaging, PET uses hypoxia-specific radioisotopes which together with ^18^F-FDG (fluorodeoxyglucose) offer a complex picture of the tumour.

## 3. The Role of PET in Hypoxia Imaging of Head and Neck Cancer

PET imaging is a valuable tool in assessing oxygenation levels in tumours for the purpose of treatment planning and also as a prognostic indicator. It was shown that the information provided by PET/CT during therapy leads to changes in treatment management in up to 40% of the cases [46].

To date, there are several hypoxia-specific PET radioisotopes in clinical use or under trial (Table 2). The feasibility and clinical adequacy of new radiolabelled isotopes are assessed by means of (1) tumour uptake and retention, (2) metabolic information supplied, and (3) patient safety. While several radioisotopes fulfill the clinical criteria as PET radiotracers, there is need for more comprehensive trials in order to distinguish in a qualitative manner between the various compounds currently used.

One category of hypoxia-specific radioisotopes is the group of radiolabelled nitroimidazole given that nitroimidazoles are known to bind selectively to hypoxic cells where they are reduced [47]. The first labeled nitroimidazole compound developed for PET detection was ^18^F-MISO ([^18^F] fluoromisonidazole) [48]. Pretreatment ^18^F-MISO uptake has been shown to be an independent prognostic indicator following treatment of head and neck cancer (high FMISO uptakes correspond to low tissue oxygen concentrations, which usually are indicative of poor response to treatment) [49]. A comparative study aiming at assessing tumour hypoxia in 38 head and neck cancer patients has been undertaken by Gagel et al. [50] using the polarographic electrode versus PET imaging with ^18^F-MISO combined with ^18^F-FDG. The group concluded that the noninvasive PET technique is valid and represents a feasible* in vivo* method for determining clinically relevant hypoxia, despite some of its limitations (such as limited spatial resolution of PET which can be a challenge when imaging small tumours).

Beside the quantitative evaluation of hypoxia-specific radiotracers, there are studies investigating the reproducibility of the tracer's uptake in the assessment of hypoxia. A study undertaken by Okamoto et al. [51] involved eleven head and neck cancer patients twice scanned with ^18^F-MISO within a 48 h interval. The difference between the two maximum standardized uptake values was 7% while the difference between the two tumour-to-blood ratios was 9.9%. The study demonstrated the reliability and reproducibility of ^18^F-MISO in regard to tumour hypoxia in head and neck carcinomas.

Another radiotracer that has been intensively studied in relation to hypoxia is ^18^F-FAZA (fluoroazomycin-arabinoside). A comparative study between ^18^F-FAZA and ^18^F-MISO undertaken on rat tumours has shown only minor advantages of the former, consisting of higher tumour to background and tumour-to-blood ratios due to more rapid clearance from blood and nontarget tissues [52].

More lipophilic fluorine-based radiotracers have also been investigated (^18^F-EF3, ^18^F-EF5); however clinical findings have shown similar results to those obtained with ^18^F-MISO. One of the newer hypoxia-specific PET agents is ^18^F-HX4 (2-nitroimidazole nucleoside analogue), which was shown to have better water solubility and faster clearance than the well-established tracers, presenting also a strong dependence on tumour hypoxia [53].

Next to fluorine, copper-based radiotracers play an important role in PET imaging of hypoxic tumour regions. Lewis et al. have published the first report on the oxygen-dependent uptake of Cu-ATSM both* in vitro* and* in vivo,* showing that hypoxic cells present with a threefold higher uptake compared to normally oxygenated cells [54].

While ^18^F-FDG is not particularly useful for the assessment of hypoxia with static PET imaging, dynamic PET offers valuable quantitative information on blood perfusion and also drug pharmacokinetics, which represents an indirect evaluation of vascular density and functionality [55].

Nevertheless, Mullani et al. [56] have shown that tumour blood flow measurements can be undertaken from the first pass of ^18^F-FDG through the tumour using a simple scan. This idea is supported by the fact that the initial large influx of the radiotracer into tissue during the first pass is delivered as a function of the blood flow to the respective tissue [55].

While more expertise is needed for dynamic PET image acquisition and interpretation, the advantage of this approach is the possibility of following over time the radiotracer's metabolism in the region of interest. This allows for a better differentiation among metabolically dissimilar areas, including differently oxygenated regions. Furthermore, dynamic PET with ^18^F-FDG gives indications not only on metabolic changes but also on vascular alterations during fractionated radiotherapy [57].

It is probably safe to suggest that all radiolabelled compounds trialed so far are valuable instruments, which assist the clinicians to decide on the most favorable subsequent treatment, thus bringing the individualized treatment planning one step closer to clinical implementation worldwide. Developing clinically robust tools for tumour hypoxia assessment would allow clinicians to choose treatment based on an individualized, scientific foundation and select those patients that would benefit from adjuvant therapies in order to sensitize the tumour to radiation. As stated by Isa et al. in a review paper published in 2006 [2]: “*there is an unmet need for biological parameters to individualize treatments.*” Several years later, while we have a larger pool of markers and biological targets available, the above statement remains valid.

## 4. Models of Hypoxia Based on PET-Imaging

Computational modelling of PET tracer dynamics is a crucial step in understanding complex individualised data acquired during PET imaging. Combined PET/CT image sets provide functional as well as spatial data from tissue regions that are, for example, highly metabolically active or relatively low in oxygen, depending on the specific radioactive tracer (or label) used. Only through the use of sophisticated models can we begin to quantitatively analyse PET tracer pharmacokinetics (PK) within* in vivo* tumours or normal tissues.

Motivation for this understanding stems from the desire of clinicians and radiobiologists to utilise specific PET data to predict tumour behaviour and responses to different treatment options, such as fractionated radiotherapy. This utilisation often requires development of further complex computational models that can read in the PET data and then apply cell line or tumour specific kinetic processes and tumour/vessel architecture to simulate proliferation and treatment induced cell death.

An initial step in interpreting and utilising complex PET data is to develop a computational model, with the aims of setting parameters with realistic values (through comparison with reported biological values) and eventually the generation of a complete virtual PET image. By these means, parameters, such as drug uptake, diffusion, and binding rates for a specific tracer, can be analysed in a model sensitivity study, with multiple-parameter solutions and associated stochastic uncertainties generated, using the real PET data as a baseline for comparison. However, model parameter values may also be patient dependent and at present the field of tracer modelling is still to reach a consensus on optimal average population values and their uncertainties.

To be case specific, the model cannot be purely based on a first principles approach using simple geometry; it requires information to spatially place important structures such as blood vessels so that the compound of interest can be accurately simulated in the blood stream and into the target tissue. This step can add imaging modality related uncertainties or other uncertainties if the data is estimated, for example, from the average vascular densities and diameters from tumour xenografts.

Two streams of reports exist on the topic of PET tracer modelling.

1. Modelling of specific tracer/oxygen dynamics—*with the aim of simulating PET images with results that are in close agreement with real PET images* [26, 58].
2. Utilisation of PET data within a separate tumour model—*with the aim of making the tumour model more specific and predictive of response to treatment* [33–35].

The subsections below are separated into the aforementioned categories, which exist due to the specific aims of the authors in either studying PET pharmacokinetics (with a strong emphasis on tumour oxygenation related tracers) or input and interpretation of voxelized PET data into computational tumour response models. Models in these two categories may utilise stochastic and/or analytical methods. The former may have final objectives of optimising PET injection and imaging sequencing protocols or investigation of the impact of acute versus chronic hypoxia on tracer uptake [26, 58], while the latter may have final aims which vary due to the treatment modality considered. However will generally have final objectives related to predicting if treatment response is enhanced with modified treatment regimes, compared to the current standard of care. In this review, the latter topic is discussed in terms of the input of specific patient PET information into cellular-scale models of tumour proliferation and radiotherapy response [33–35].

### 4.1. Modelling PET Pharmacokinetics (PK)

Modelling of the transport of drug molecules through tissue, or “pharmacokinetics,” applied to the field of PET radioactive tracers, has been studied for a number of decades. For the modelling of tracers that preferentially reduce and bind in the presence hypoxia or anoxia (i.e., a lack of or no presence of oxygenation), early 2- and 3-compartmental analytical model work was published from approximately the mid-nineteen seventies and into the first years of the twenty-first century [59–61].

Compartmental modelling in this respect refers to the spatial location (it's “state” may also be specified) of the tracer as it moves through tissue after injection; that is, the tracer could exist: (1) in a free state within vessels (within blood plasma), (2) in the interstitial space, or (3) in the intracellular space, (i) a free or (ii) a bound (reduced/phosphorylated) state. Note that the 2-compartment models neglect the second step. The tracer may be modelled to move from (1) to (2) via diffusion and/or interstitial fluid convection, while movement from (2) to (3) requires active transport, facilitated diffusion, and/or passive diffusion [25, 28].

Tabulated above is a selection of recent reports, where modelling groups have aimed to predict and better understand PET imaging data for specific tracers (Table 3). These groups may have also performed simultaneous histological or secondary imaging tests to compare with their primary PET data set and model results, to validate the placement of well vascularised, proliferative, or hypoxic tumour subvolumes [25, 28, 29]. Secondary imaging has in some cases also enabled realistic (and specific for the tissue being studied) vessel maps to be incorporated into models for particular tumour cell lines [30]. This allows the tracer and also oxygen transport to be simulated in as close a manner (spatially) as possible to the real situation. In general, model predictions may be in the form of 2-dimensional virtual PET images which can be compared to real PET images, or in the form of parametric solutions for the mathematical model utilised, that is, the best fit and statistical analysis of all compartment model coefficients/parameters.

To summarise the contents of Table 3, analytical compartment model work has been extended in recent years to specific and relevant PET tracer molecules, with statistical analysis of the best fit of the model parameters being found to be in good agreement with biological interpretation of the parameters (e.g., the diffusion rate constant of the tracer through capillary walls) and with the generated virtual PET images correlating well with patient and animal model PET image sets. These models show that due to the dynamic action of the tracer molecules, multiple or dynamic PET sequences are required to best interpret PET data (e.g., to assess hypoxic tumour regions). Early scans tend to indicate blood vessel tracer presence, whereas later scans are required to gain information about the desired more “final” end points of these tracers in the target cells. However, specific tracer PK information is vital. Modellers as well as clinical PET researchers may have analysed their results using different terms, commonly, time-activity-curves [25], standard uptake value (SUV) with the mean or maximum values analysed for each voxel [17], or tumour-muscle (TMR) or tumour-blood (TBR) signal ratios [62]. The latter method normalizes results for each individual to base-line levels within their own normal tissue.

Problems in the comparison of virtual PET results with real data inevitably arise due to noise in the images and the absence of tracer in tissue regions in which the tracer has difficulty in reaching, for example, very hypoxic/necrotic tissue. As a consequence, the functions of tracer concentration (at any time point) required in models are not likely to correspond linearly with tissue oxygenation. Rather, the highest signal is likely to emanate from regions of low to moderate hypoxia, where transport and binding probability of the tracer is the highest compared to very low oxygenated regions where binding is possible but vessel integrity and density is insufficient. The PET tracer utilised may also have multiple biological pathways and reactions; for example, Cu^64^[ATSM] has been shown to correlate well with hypoxic tumour data due to its electrochemical properties, however “…*the precise mechanism of Cu-ATSM accumulation remains largely unknown, as Cu-ATSM accumulation occurs under normoxic conditions and is strongly influenced by genetic characteristics that are independent of pO*_*2*_ *status*” [29].

Common assumptions made in PK and O_2_ diffusion models due to limitations of complexity, time, computing power, and available parametric data include the following.

1. Homogeneous cellular density, diffusion, and consumption coefficients, which is not the case in a heterogeneous tumour.
2. The use of static histological data used to generate vessel maps and hence the assigning of O_2_ in 2- or 3-dimensional maps does not reflect acute hypoxic dynamics, which can be overcome by specifically modelling O_2_ kinetics [30] with realistic temporal fluctuations in O_2_ supply and realistic cellular O_2_ consumption [28].
3. The lack of machine specific corrections (dead time, decay rate, noise, scatter, attenuation correction, and time-of-flight) and reconstruction related random and systematic uncertainties, considered however by some groups [28, 33] and specifically investigated in terms of attenuation correction based on X-ray CT data [63] or segmentation of emission images and in terms of partial volume effects [64].

Neglected in Table 3 are reports from authors addressing oxygen and/or tracer dynamics using multimodality imaging techniques without a “modelling” approach (see Section 3). These reports can add valuable data in terms of correlations between anatomy, tracer, and oxygenation in space and time, for example, the work of Cho et al., who have combined PET (F^18^[MISO]), MR, H&E, pimonidazole, and F^18^ autoradiography techniques in a prostate xenograft tumour model to analyse necrosis, hypoxia, and well perfused tissue and to confirm the validity of the different imaging modalities in identifying these tumour subvolumes [37]. Cho et al. [37] found that combined hypoxia and perfusion data could predict outcome but could neither alone correlate well with regions of perfused tissue (as indicated by early PET uptake).

### 4.2. Utilisation of PET Data in Treatment Response Models

As the previous section has shown, there are many factors that influence final PET image voxel intensities. These factors reduce down to being related to both tracer pharmacokinetics and specificity or machine intrinsic spatial resolution limits. When desiring to take this data and use it for input into computational tumour models, these factors must also be of paramount concern. The tumour model, if at the cellular-scale, will require a suitable scaling paradigm to convert data at the mm scale down to an approximate 10–50 *μ*m scale and also method of converting the PET signal intensity assigned to each tumour voxel or “cell” into an actual parameter value, such as pO_2_ if, for example, oxygenation data has been the intention of the PET scan.

Stochastic methods utilising probability distributions may be useful for both of these tasks. Models capable of simulating tumours at the cellular level with real tumour-like proportions are challenging, hence limited number of papers reviewed. This topic is however at a stage of expansion and it is foreseen that more groups will report of their modelling experiences in the field of PET data tumour response modelling within the present decade. This will be aided by not only computing power increases, but also the demand by clinicians and the public to use currently, or soon-to-be, available individualised PET data for specific (not only region of interest contouring) purposes during treatment planning.

Two reports from stochastic tumour modelling groups are summarised in Table 4. These groups have developed models of varying complexities, including treatment response modelling, with discussions about how the previously presented limitations and issues have been considered.

To expand upon the work that has been performed to estimate modified treatment responses, Table 5 has been included to further highlight efforts that have been made to consider tumour model predictions within clinical radiotherapy treatment planning systems (TPS) dose distributions, where the plan has either been delivered to real patients or the distributions analysed for their feasibility in terms of deliverability and toxicity.

A thorough overview of the process of gaining information from PET/CT and using it to optimise pre- and midtherapy planning processes was published in 2013 [65]. The authors concentrate on how hypoxia related information can be obtained and used in the planning process, from interpretation of PET data to final planning and prescriptions for radiotherapy. Dose painting (DP) methods, for example, methods to “redistribute dose” to the most radioresistant HTVs or escalating/boosting the dose to HTVs above the standard uniform dose prescription, are discussed along with many of the current challenges that require more research and validation. Indeed, modern IMRT techniques are confirmed here as being capable of delivering complex nonuniform dose gradients with a resolution similar to that of PET images which could in theory deliver a gradient of prescription doses to subvolumes with different severities of oxygen deprivation.

Geets et al. remind us of the difficulties faced in implementation arising not only from intrinsic noise, blur, and partial volume effects in PET data, but also in the use of a suitable tracer that is specific and can reach the hypoxic tumour environment (i.e., low probability of being bound/reduced due to other microenvironmental factors such as low pH) and the use of suitable scan time protocols. References are provided from groups reporting workable solutions to uncertainties arising due to the image acquisition itself; however, as the reports in Table 3 confirm, the latter issues may not be easily overcome without future tracer specificity research and scanning at multiple time points after injection. F^18^[FAZA] is mentioned as showing promise as good hypoxia tracer; however all tracers did [65] and continue to have issues, with no compound standing out as superior.

This report also discusses the issues of converting oxygenation information into radioresistance estimates and hence prescription requirements, in order to achieve increased tumour control. These are nontrivial challenges, as both functions of converting pO_2_ to cell death probably, that is, radiosensitivity, or cell death to tumour control are nonlinear and dependent on patient specific parameters. The authors remind us that chronic hypoxia remains the focus of the discussed techniques, as opposed to acute/transient hypoxia which may alter in oxygenation faster than the time a PET scan can be acquired and analysed (or radiotherapy planned/delivered). Logistical noteworthy challenges are discussed, for example, the set-up accuracies required, volume expansion protocols for adding margins onto regions of interest nonuniform dose prescription, and how often to rescan during therapy to balance workload of scanning and replanning with the timing of substantial biological change and hence improvement in therapeutic ratio if the plan is altered.

## 5. Conclusions

Tumour hypoxia remains one of the major causes of treatment failure in head and neck cancer. By promoting angiogenesis as well as distant metastases, hypoxia becomes an important treatment target. In order to increase the therapeutic ratio it is crucial to identify and to quantify the hypoxic subvolumes. To date, PET-based molecular imaging is the most commonly employed technique applied for this purpose.

The use of computational models for treatment assessment and prediction is fast growing. Whether simulated or actual PET data has been applied to a tumour response model, overall the current literature suggests that targeting radioresistant hypoxic tumour subvolumes using complex dose gradients or even simpler boost doses is feasible using modern radiotherapy techniques. Research has also shown that toxicity need not be compromised if careful planning is performed, with a possible solution being not to increase the integral dose but rather decrease the prescription to well oxygenated regions and increase it to chronically hypoxic areas. This is still to be confirmed in randomised clinical trials.

As it stands currently, all the signs point to hypoxia dose painting as being feasible to tackle notoriously hypoxic tumours, such as head and neck carcinomas. To further improve, the field moves into requiring individual tracer pharmacokinetic information/analysis so that PET data can be accurately interpreted and then utilised appropriately to predict optimal treatment plans and overall outcome improvement.

Health institutions will need to encourage and support multidisciplinary research and prioritize resources to make the use of functional PET information feasible, as assessing the dynamic changes of the tumour characteristics at a number of time points after PET injections and then at multiple intervals throughout radiotherapy will always inevitably be highly resource intensive. This will be especially true during the learning phase as this technique is translated from research into the routine clinical environment. Computational pharmacokinetics and tumour models will be vital in this translation process.

## Figures and Tables

**Table 1:** Techniques for tumour hypoxia evaluation/measurement.

Technique	Characteristics
Polarographic electrode (Eppendorf oxygen electrode)	Direct and invasive technique involving a fine-needle electrode (cathode) for tumour hypoxia measurement. The current between the cathode and the reference electrode is directly proportional to tissue pO_2_.

Cryospectrophotometry	Indirect, histomorphometric assay of oxygen levels in tumour vasculature assessed on frozen tissue samples.

Microvessel density (angiogenesis assessment)	Indirect way to assess hypoxia using immunohistochemical techniques for counting blood vessels that were previously labeled with endothelium-specific markers.

DNA strand break assay (comet assay)	Indirect way to assess tumour hypoxia through DNA strand breaks after radiation exposure and fluorescent staining, based on the fact that oxic cells get more damage than hypoxic cells. The DNA fragments detached from the nucleus resemble the tail of a comet.

Endogenous hypoxia markers	Indirect method to evaluate the hypoxic fraction in tumours. Hypoxia inducible factor (HIF)-1 alpha, glucose transporter 1 (GLUT 1), and carbonic anhydrase 9 (CA 9) have been identified as proteins, which under hypoxic exposure induce the transcription of several genes.

Exogenous hypoxia markers	Indirect method to evaluate tumour hypoxia (using biopsies). Exogenous markers are nitroaromatic compounds (pimo-, miso-, eta-nidazole) which selectively bind to hypoxic cells.

Oximetry with electron paramagnetic resonance	Noninvasive and direct method to quantify pO_2_ in tissue using stable nitroxides that interact with molecular oxygen.

Blood oxygen level-dependent magnetic resonance imaging (BOLD MRI)	Noninvasive method for evaluation of hypoxia through correlation of BOLD signals with pO_2_.

Positron emission tomography	Noninvasive and direct method to evaluate hypoxia via injection of hypoxia-specific radiotracers.

**Table 2:** Trials involving hypoxia-specific PET imaging in head and neck cancer over the last 10 years.

Radiotracer	Reference	Trial and aim	Results
^ 18^F-MISO	Rischin et al. 2006 [16]	*TROG randomized trial (45 patients)* To determine the association between tumour hypoxia, treatment regimen, and locoregional failure in advanced HNC	^ 18^F-MISO-detected hypoxia is associated with high locoregional failure in patients not receiving tirapazamine.
	Kikuchi et al. 2011 [17]	*Clinical study (17 patients)* To evaluate the role of ^18^F-MISO as a predictor of treatment outcome in HNC	Local control after radiotherapy was significantly lower in patients with high uptake than in those with low tracer uptake. Pretreatment scan with ^18^F-MISO may predict treatment outcome.

^ 18^F-FAZA	Souvatzoglou et al. 2007 [18]	*Phase I trial (11 patients)* Feasibility of ^18^F-FAZA for hypoxia imaging in HNC patients	Feasible for clinical use and offers adequate image quality for hypoxia assessment.
	Postema et al. 2009 [19]	*Phase I trial (9 HNC patients out of 50 overall cancer patients)* To demonstrate the safety and biodistribution pattern of ^18^F-FAZA in patients with HNC, lung cancer, gliomas, and lymphomas	Clear uptake of ^18^F-FAZA was observed in 6 out of 9 HNC patients; good imaging properties; good tumour-to-blood ratio. Promising agent for hypoxia imaging in HNC.

^ 18^F-EF3	Mahy et al. 2008 [20]	*Phase I trial (10 patients)* To assess the pharmacokinetics, biodistribution, and metabolism (324 MBq versus 1,134 MBq)	Uptake and retention in tumour was observed; no difference between the radioactivity groups; no side effects; safe and feasible.

^ 18^F-EF5	Komar et al. 2008 [21]	*Phase I trial (15 patients)* To determine the optimal PET protocol for ^18^F-EF5 as a hypoxia imaging agent	Initial ^18^F-EF5 uptake is governed by blood flow; later phase uptake is hypoxia specific (optimal detection time is 3 h after injection); warranting more study.

^ 18^F-HX4	Chen et al. 2012 [22]	*Phase I trial (12 patients)* To evaluate the feasibility of HX4 compared with ^18^F-MISO	HX4 possibly has higher sensitivity and specificity and shorter injection-acquisition time (1.5 h) than ^18^F-MISO

Cu-ATSM	Minagawa et al. 2011 [23]	*Phase I/II trial (17 patients)* To evaluate the relationship between ^62^Cu-ATSM tumour uptake and chemoradiotherapy	^ 62^Cu-ATSM SUV_max_ greatly differed between patients with and without residual disease. ^62^Cu-ATSM could be a predictor of tumour response to treatment.
	Grassi et al. 2014 [24]	*Preliminary prospective study (11 patients)* To assess the efficacy of pretreatment ^64^Cu-ATSM as a prognostic factor and its role as a marker of disease progression	^ 64^Cu-ATSM showed high sensitivity but low specificity in predicting response to chemoradiotherapy. There were no differences between early and late scans.

HNC: head and neck cancer.

**Table 3:** Models from the literature that simulate the pharmacokinetics (PK) of PET tracers to predict and analyse PET scan images.

Reference	Modelling Methods	Details and Outcomes
(Kelly and Brady 2006) [25]	2-compartment F^18^[MISO] PK model with reversible binding, with transport via diffusion only. 2-dimensional analytical spatiotemporal model.	Michaelis-Menten techniques were used to model the conservation of O_2_ and cap consumption in oxic tissue (pO_2_ dependent equation). Randomly angled/oriented vessels, temporal dynamics modelled by changing vessel pressure and hence flow. Hypoxic tissue: gradual increase in activity then an accumulation curve. Oxic tissue: activity follows plasma levels then accumulation curve seen at later stages. Late slope of TAC curve indicated hypoxia while the beginning represented local vascular supply. Results compared to pimonidazole stained tumour sections from clinical colorectal cancer data.

(Wang et al. 2009) [26]	Iterative stochastic optimisation algorithm to delineate acute and chronic hypoxia in sequential F^18^[MISO] and FDG PET in 2-dimensional image maps, with comparisons to HNSCC clinical data.	Simulated images (known hypoxic regions) as well as sequential PET Data from 14 male HNSCC patients analysed assuming chronic (Gaussian histogram of number of voxels versus SUV) hypoxia remained constant while acute hypoxia (Poisson histogram) was varied. Normalisation methods forced the volume of hypoxia to be the same in both time-point scans; however the location of acute hypoxia varied. Image registration and resolution issues are discussed. Model predicted Gaussian chronic hypoxia distributions well in the generated images (*r* ^2^ = 0.93). Good fit found (13/14 cases), with acute hypoxia described well by a Poisson curve (11/14 cases) with an average of 34% (acute hypoxic volume). Suggested a third scan to increase temporal hypoxia information. 4 mm PET pixel size issue accounted for using power law distribution of chronic versus acute hypoxia within each pixel.

(Bartlett et al. 2012) [27]	Two varieties of 2-compartment, 3-rate-constant models applied to F^18^[MISO] PET images of human prostate tumour xenografts in rats.	One model constrained kinetic parameters *k*_1_ and *k*_2_ to be equal while the other did not. Intratumoural pO_2_ was assessed using a robotic driven probe in tumour versus plasma regions of the animal's tumour mass. Pimonidazole and perfusion Hoechst 33342 staining also analysed. Kinetic voxelised modelling (of parameter *k*_3_) identified hypoxia with greater accuracy than tumour-to-plasma ratios. Constraining *k*_1_ to equal *k*_2_ during fitting was effective in controlling noise in the trapping rate constant, *k*_3_, without introducing bias. No obvious pO_2_ cutoff for isolating hypoxic and nonhypoxic volumes (3.4 mm Hg applied) however noise of approx. 0.7 mm Hg in measurement technique.

(Gu et al. 2012) [28]	3-compartment F^18^[FLT] PK model (3 rate constants) applied to a separate GBM growth model utilising spatial MR data and considering invasion, hypoxia, necrosis, and angiogenesis.	Voxels assigned “cell density” values with hypoxic versus oxic percentages (e.g., 70 versus 30%) generated. Model simulated the dynamic clinical-scale imaging process in terms of noise and reconstruction uncertainties of PET. Clinical GBM patient data used for comparison, with patient specific virtual PET scans generated with no statistical difference to real hypoxic tumour image sets. Model could predict and distinguish hypoxic cell hyperactivity versus hyperdensity on the PET image.

(McCall et al. 2012) [29]	TACs derived from mean tissue activity concentration functions for Cu^64^[ATSM] (Ct) in HNSCC and muscle and compared to venous input functions (Cp).	Tracer dynamics studied in HNSCC (FaDu) xenografts in rats and analytical parameters of the model fitted to generated results matching real PET data. Influx-constants (Ki) calculated by analysis of Patlak plots of Ct/Cp ratios versus normalized time integrals of Cp. PET mean data analysed from 1 min up to 18 hours after injection. Distribution volumes (*V*_*d*_) calculated. High tumour to muscle uptake ratios found (4 : 1 tumour to muscle ratio at 20 min). No Cu^64^[ATSM] correlation to pimonidazole hypoxia staining (early or late). Cu dynamics are not only pO_2_ dependent, more study recommended. Early uptake of tracer in tumour at 1 min found followed by slower but steady increase, while muscle signal increased quickly then plateaued. Wash out rates in tumour and normal tissue difficult to define.

(Monnich et al. 2013 [30], M$\ddot{\text{o}}$nnich et al. 2011) [31]	O_2_ kinetic and F^18^[MISO] tracer PK model simulating 2-dimensional virtual PET maps, based on blood vessel maps from human HNSCC xenografts stained for endothelial structures	Xenografts were utilised to derive 2D vessel maps (~3% vascular fraction) and an explicit pO_2_-dependent binding rate, *K*(*P*). Oxygen and tracer flux across vessel walls, *J*_*T*_, assumed proportional to the concentration differences on the intra- and extravascular side. Tracer moved via diffusion. Irreversible binding rate modelled as dependent on pO_2_ only_. _Nonlinear Michaelis-Menten oxygen consumption versus pO_2_ tension. Individual time-point data did not show correlation with real data (2.5 mm Hg threshold for each voxel with median pO_2_ in each voxel assessed); however, ratios of 0–15 min versus 4-hour data had significant outcomes. Four-hour data did correlate but not as well as ratio data. From 2011: binding versus pO_2_ function described with steep initial increase (<0.5 mm Hg). Simulated local TACs share characteristics with clinical PET TACs hence it may be possible to measure perfusion from early dynamic PET. Alternative tracer dynamics (faster clearance) also simulated with earlier time point PET scans predicted optimum, although free-tracer signals limit earlier time feasibility.

(Liu et al. 2014) [32]	F^18^[FLT] 2- and 3-compartment PK models compared for HNSCC clinical PET images, incorporating diffusion as well as convection transport of the tracer.	A comprehensive statistical analysis of the PK model is reported. “EM-BIC” clustering methodology described, and model used to analyse raw PET images and reduce noise and hence uncertainty in the rate constant parameters derived. Model results compared to 10 × 1-hour dynamic HNSCC clinical PET data sets, with the 3-compartment (6 rate constants) “3C6K” model best fitted patient data.

[TAC: time activity curve; HNSCC: head and neck squamous cell carcinoma; GBM: glioblastoma multiforme; PK: pharmacokinetic; pO_2_: partial pressure of oxygen; SUV: standard uptake value].

**Table 4:** Stochastic tumour models utilising PET oxygenation data to predict the efficacy of nonstandard treatment solutions.

Reference	Modelling methods	Details and outcomes
(Toma-Daşu et al. 2009) [33]	F^18^[MISO] and Cu[ATSM] distribution functions modelled in a 10^8^ cell tumour growth and O_2_ transport model followed by uniform or central boost RT.	Tracer binding versus pO_2_ functions used (higher uptake at intermediate O_2_ for Cu) to generate tracer uptake maps for each tracer on a 2D slice of heterogeneous spherical tumour. Convolution function used to describe finite resolution of the imaging modality. Local temporal changes in cellular O_2_ accounted for. Virtual image maps generated to predict LQ survival and Poisson tumour control using 2 different circular dose distributions with central boost doses. Redistribution of dose (same integral dose but hotter in the centre) was possible for each tracer without decreasing the target tumour control (90%).

(Titz and Jeraj 2008, Titz et al. 2012) [34, 35]	Simulating effects of antiangiogenic treatment using F^18^[FDG], F^18^[FLT] and Cu^61^[ATSM] PET data in a tumour proliferation and therapy model (2008—where RT is modelled as the treatment modality) with an added vascular and PET/drug PK/PD component (open 2-compartment) (2012).	*BvMb* plasma concentration-time-profiles *cbev(t)* utilised, with model parameters adapted to population-based values (e.g., MVD to determine *BvMb* PD). A linear relationship between VEGF expression and endothelial cell (EC) proliferation used. Nonnormal distributions manipulated the raw data O_2_ PET data (~4 mm pixels) for cellular level input to generate 2D oxygenation maps considering multiple diameters and angles of the vessels. 8 HNSCC PET scans used (phase 1 trial data), before and after RT for input and comparison to model predictions. A decrease in SUVs (i.e., reduction in vasculature) after *BvMb* agreed with follow-up PET. Increase in hypoxia due to *BvMb* observed, peaking at week 2 after treatment, but decreasing with increasing baseline levels of hypoxia and increasing CCT. Due to pO_2_ and proliferation interdynamics, simulations could provide estimates of optimal drug administration times (i.e., every 2 or 3 weeks). Expansion planned for the use of voxel-based kinetic parameters to model drug uptake more precisely and vessel “remodelling” in response drugs.

[PD: pharmacodynamic; CCT: cell cycle time; BvMb: bevacizumab].

**Table 5:** Application of model predictions to clinical radiotherapy dose distributions to increase tumour control in hypoxic tumours.

Reference	Treatment/Model Methods	Details and Outcomes
(Thorwarth and Alber 2008) [36]	F^18^[MISO] PET/CT performed on 15 HNC patients, with mid-RT scan after 20 Gy and with total dose of 70 Gy. DP strategies investigated.	Hypoxia and well as perfusion parameters combined could predict for RT outcomes, but neither alone (similar to study by Cho et al. 2009) [37] Model was calibrated using hypoxia and perfusion outcomes from this patient set and was designed to be used to predict optimal dose escalation factors to radioresistant HTVs. DP found feasible without increased toxicity to normal tissues.

(Choi et al. 2010) [38]	IMRT dose escalation to the HTV (from 2.4 to 2.6–3.6 Gy/30 fractions) planned for 8 HNSCC patients after F^18^[MISO] PET/CT (4 hours post injection). ECLIPSE TPS and 6 MV X-rays beams utilised.	Tumour/cerebellum activity ratio of 1.3 used as a cut-off value for HTV definitions. Dose escalation to at least 2.6 Gy to the HTV found feasible for 6/8 patients, where the HTV received a total of 78 Gy, without increasing normal tissue doses.

(Toma-Dasu et al. 2012) [39]	IMRT optimisation performed using a research TPS to plan dose distributions for various scenarios of HTV evolution during RT. Data from 7 HNSCC patients after F^18^[MISO] PET/CT (120–160 min post injection) applied. HTV aim (dynamic pO_2_ case) of increasing dose from 60 to 77 Gy.	PET signal to uptake (and hence pO_2_ and then radiosensitivity) data conversion used a maximal pO_2_ level of 60 mm Hg and analytical formula. Model provides an objective method to set minimum doses to hypoxic regions to counteract increased radioresistance in individual tumours, without comprising tumour control, that is no decreasing non-hypoxic volume doses below current clinical doses.

[DP: Dose Painting; HTV: Hypoxic Target Volume; IMRT: Intensity Modulated Radiotherapy; TPS: Treatment Planning System; RT: Radiotherapy].
